# Health evaluation and referral assistant: a randomized controlled trial to improve smoking cessation among emergency department patients

**DOI:** 10.1186/s13722-015-0045-2

**Published:** 2015-11-05

**Authors:** Edwin D. Boudreaux, Beau Abar, Brianna Haskins, Brigitte Bauman, Grant Grissom

**Affiliations:** Departments of Emergency Medicine, Psychiatry, and Quantitative Health Sciences, University of Massachusetts Medical School, LA-189, 55 Lake Avenue North, Worcester, MA 01655 USA; Departments of Emergency Medicine, Psychiatry, and Public Health Sciences, University of Rochester Medical Center, Rochester, NY USA; Department of Emergency Medicine, University of Massachusetts Medical School, Worcester, MA USA; Department of Emergency Medicine, Cooper Medical School of Rowan University, Camden, NJ USA; Polaris Health Directions, Inc, Langhorne, PA USA

**Keywords:** Tobacco, Brief intervention, Emergency medicine, Facilitated referral, Tobacco treatment, Tobacco screening

## Abstract

**Background:**

Computer technologies hold promise for implementing tobacco screening, brief intervention, and referral to treatment (SBIRT). This study aims to evaluate a computerized tobacco SBIRT system called the Health Evaluation and Referral Assistant (HERA).

**Methods:**

Smokers (n = 421) presenting to an emergency department were randomly assigned to the HERA or a minimal-treatment Control and were followed for 3 months. Analyses compared smoking cessation treatment provider contact, treatment initiation, treatment completion, and smoking behavior across condition using univariable comparisons, generalized estimating equations (GEE), and post hoc Chi square analyses.

**Results:**

HERA participants were more likely to initiate contact with a treatment provider but did not differ on treatment initiation, quit attempts, or sustained abstinence. Subanalyses revealed HERA participants who accepted a faxed referral were more likely to initiate treatment but were not more likely to stop smoking.

**Conclusions:**

The HERA promoted initial contact with a smoking cessation provider and the faxed referral further promoted treatment initiation, but it did not lead to improved abstinence.

Trial registration: ClinicalTrials.gov number NCT01153373

## Background

Each year tobacco use kills 430,000 Americans for an average of over 5 million years of potential life lost annually [1–4]. Smoking cessation is a cost-effective disease prevention strategy that has shown immediate and substantive health benefits in those who successfully quit [3]. While much attention has focused on primary care as a setting to promote smoking cessation, hospital emergency departments (EDs) are uniquely positioned for tobacco intervention efforts as well. The prevalence of tobacco use among ED patients exceeds the national average [2, 5, 6], with a 2014 study reporting an overall prevalence of more than double the national average [5]. Furthermore, disadvantaged and underserved populations, such as minority patients, underinsured, and low income households, rely disproportionately upon EDs for primary care services [7]. With over 130 million visits annually, ED originated tobacco control efforts have the potential for substantial impact on public health by effecting change in hard to reach populations [7, 8].

This potential was recognized by the Robert Wood Johnson Foundation which recommended implementation of routine screening, brief intervention, and referral to treatment (SBIRT) for tobacco in the ED setting, and strongly encouraged research to develop tobacco interventions specific to the needs of EDs [7]. Additionally, a joint statement by the American College of Emergency Physicians and the Emergency Nurses Association recommended that ED healthcare providers universally screen and counsel tobacco users seeking care in the ED. They proposed using a brief Ask, Advise, Refer model, which consists of Asking about tobacco use, Advising patients to quit, and Referring patients to specialized treatment for tobacco use [2, 3]. The Ask, Advise, Refer model has since been adopted by researchers in their work to refine an effective ED-appropriate strategy for tobacco SBIRT [9, 10].

While much literature supports the utility of tobacco SBIRT for the ED population [2–7, 9–11], questions still remain regarding the most effective and appropriate SBIRT protocol. Factors likely to impede clinician intervention include competing time demands, lack of specialized behavioral health training, and a mission centered on acute rather than chronic medical care [6–8]. To maximize successful implementation, procedures should be convenient, brief, require minimum specialized training, and focus on connecting patients to outpatient resources after the ED visit. Traditional strategies typically do not meet all of these requirements and result in inconsistent adoption [12]. Behavioral intervention technology advancement has allowed for the development of computer-assisted SBIRT models which aim to reduce interruptions in clinical care and reduce clinician burden without sacrificing effectiveness [8].

The objective of this study was to test a new web-based program to facilitate tobacco SBIRT called the Health Evaluation and Referral Assistant (HERA). The HERA was patterned after face-to-face SBIRT models and is self-administered by patients on a computer during their medical visit. This study tested the hypotheses that the HERA would result in greater outpatient smoking cessation treatment initiation and increased smoking cessation over three months post-visit as compared with a minimal intervention control condition.

## Methods

A full description of the HERA development and RCT methods were previously reported [12, 13]. Although the HERA assesses for and refers patients to treatment for tobacco, alcohol, and illicit drug use, only results pertaining to tobacco are reported in this paper. Subsequent papers will address the other substance classes. This clinical trial was registered with ClinicalTrials.gov as the Dynamic Assessment and Referral System (ClinicalTrials.gov Identifier: NCT01153373).

### HERA

*Assessment* The HERA uses the two-item Heavy Smoking Index (HSI) to assess nicotine dependence [14]. Age of first smoking cigarettes regularly, use of other forms of tobacco, such as cigars or smokeless tobacco, and readiness to change were also assessed. Participants who expressed interest in quitting were asked when they would like to quit (within the next 30 days, Within the next 6 months, more than 6 months from now), and if they would like help connecting with a smoking cessation counselor or treatment program (Yes, No).

A checklist of psychiatric diagnoses was used to document psychiatric history [none, anxiety or panic attacks or post-traumatic stress disorder (PTSD), depression or bipolar disorder, schizophrenia or schizoaffective disorder, anorexia or bulimia, attention deficit disorder (ADD, ADHD), other]. Participants were also screened for depression using the Patient Health Questionaire-2 (PHQ-2) [15].

*Report generator* Two reports using the assessment data were produced, and are described in detail by Boudreaux et al. [12, 13]. The Healthcare Provider Report is a summary of the assessment and was reviewed by the patient’s treating physician. The Patient Feedback Report consisted of three sections: (1) the Face Sheet, which included an overview and tailored tobacco referral list, (2) the Patient Assessment Summary, which provided personally tailored feedback related to the patient’s tobacco use, and (3) the Motivation Toolkit, which consisted of several worksheets rooted in motivational interviewing [16] and the transtheoretical model [17].

*Referral generator* The referral generator draws upon a library of smoking cessation treatment services maintained by Polaris Health Directions, Inc. The library is used to generate personally tailored referral lists containing free and fee-for-service treatment options, including free state quitlines, and to send dynamic referrals. A dynamic referral is a faxed referral to a “best match” facility based on the individual’s ZIP code, insurance, and desire for telephone or in-person treatment. If accepted by the patient, the dynamic referral was faxed by the HERA and included a brief assessment summary and the patient’s contact information. The participating services agreed to contact the individual within 48 h of receiving the referral to perform an initial evaluation and explore treatment options.

### Procedure

Patients were enrolled from four EDs (see Table 1) during 8 AM to 7 PM, with shifts representing all days of the week. Research assistants (RAs) approached consecutive adult patients at their bedside. All patients who smoked within the past 30 days and 18 years or older were considered. Patients with risky alcohol use or illicit drug use, including those who currently demonstrate tobacco dependence, were enrolled into the larger trial and examined separately due to the overarching goal of intervening on the most salient/emergent substance-related concerns. The current paper focuses only on tobacco users who did not abuse alcohol or use drugs. Exclusion criteria included severe illness or distress, cognitive insufficiency, in state custody or restraints, being held involuntarily, and language barriers. Patients were enrolled regardless of whether they were admitted or discharged; the study components, including baseline and intervention, were completed prior to leaving the ED. Participants were randomized to either the intervention or control condition by a random number generator from the Java programming language standard library embedded within the HERA. Immediately following discharge/transfer from the ED, the enrolling RA completed a short interview with the participant, either in person before he or she left the ED or by phone within 48 h (post-visit interview). A trained RA unaffiliated with the performance sites contacted all participants by telephone at 4 and 12 weeks following the ED visit to assess tobacco treatment initiation and to re-assess tobacco use. Biological verification of abstinence was not obtained. The study was approved by the Institutional Review Boards for all performance sites. All participants gave their informed consent and signed a written consent form prior to their inclusion in the study.

**Table 1:** Site characteristics

Type	Annual volume	Location	Race/ethnicity
Academic, urban	90,733	Worcester, MA	W 82 %, H 11 %, B 4 %
Community, urban	47,364	Worcester, MA	W 74 %, H 14 %, B 9 %
Community, suburban	23,217	Marlboro, MA	W 80 %, H 15 %, B 3 %, U 2 %
Academic, urban	59,482	Camden, NJ	W 35 %, H 20 %, B 45 %

*W* white, non-Hispanic, *H* Hispanic, *B* black, *U* unknown, *MA* Massachusetts, *NJ* New Jersey

### Study conditions

Intervention and control conditions were treated the same in all respects related to study procedures. Those in the intervention condition (HERA) (1) were offered a dynamic referral, (2) their treating physician was given the Healthcare Provider Report, and (3) the patient was given the Patient Feedback Report, which included a personally tailored referral list. Those assigned to the minimal intervention control condition (Control) completed the HERA assessment but were not offered the dynamic referral, and the reports were not made available to either the healthcare provider or the patient. However, Control group patients did receive a standardized, printed list of local smoking cessation treatment providers and included the toll-free tobacco quitline 1-800-QUIT-NOW.

### Blinding

The RA performing the outcome assessments was partially blinded. Because the HERA is heavily focused on the referral process, and different patients received different types of referrals, the follow-up questions were keyed to the referral type received at baseline (printed list vs. dynamic referral) to avoid confusion. Despite blinding efforts, the nature of the questions revealed some information about the group assignment if the participant had accepted a dynamic referral. For example, only patients who chose a dynamic referral were asked whether they had been contacted by a smoking cessation counselor.

### Measures

*HERA* The HERA assessment was described under methods/assessment module.

*Post*-*visit interview* Immediately following discharge/transfer from the ED, the enrolling RA completed a short interview to ascertain if the treating clinicians provided tobacco counseling, education materials, or referrals for tobacco treatment. Chart review was not used because of unreliability associated with documenting tasks such as counseling.

*Follow*-*up assessment* All participants were asked if they had made initial contact with a smoking cessation counselor or treatment program (treatment contact), completed an initial assessment (treatment initiation), attended any additional treatment sessions beyond the initial assessment (treatment engagement), and completed treatment (treatment completion). Any of these milestones could have occurred in person or by telephone. The RA interviewed the participant by phone to assess a self-reported quit attempt defined as intentional abstinence for 24 h, efforts to decrease tobacco use, the HSI, and sustained abstinence since the ED visit.

### Data analyses

In addition to univariable comparisons, the primary analysis consisted of a series of generalized estimating equation (GEE) analyses comparing the control and intervention conditions on outcomes of interest (tobacco treatment provider contact, treatment initiation, tobacco use) during the 4 and 12 week follow-up. When GEE models were statistically significant, post hoc Chi square analyses were used to provide greater context for the observed findings. These models were unconditional (i.e., included no covariates) due to random assignment equating groups on potential confounding characteristics. A series of planned Chi square analyses were also performed for outcome data available at a single time point (e.g., ED counseling, treatment completion).

Analyses were supplemented by examining differences across individuals in the control condition versus those in the intervention condition that accepted a dynamic referral and those in the intervention (dynamic referral) condition that declined a dynamic referral (tailored list only). These supplemental models included baseline HSI and readiness to quit as covariates to account for preexisting differences between individuals who accept a referral and those who do not. All GEE models were performed first using multiple imputation with 20 datasets to account for missing data at each of the follow-ups. These models were then recapitulated under intention-to-treat principles (i.e., non-retained participants provided with the least favorable outcome), and the patterns of statistical significance of primary predictors and covariates were identical. Observed frequencies were presented in each table, with the percentages representing intention-to-treat values. An alpha level of 0.05 was used to indicate statistical significance for all comparisons, and all analyses were performed using SPSS 22 (IBM, 2012).

## Results

### Preliminary analysis

**Fig. 1:**
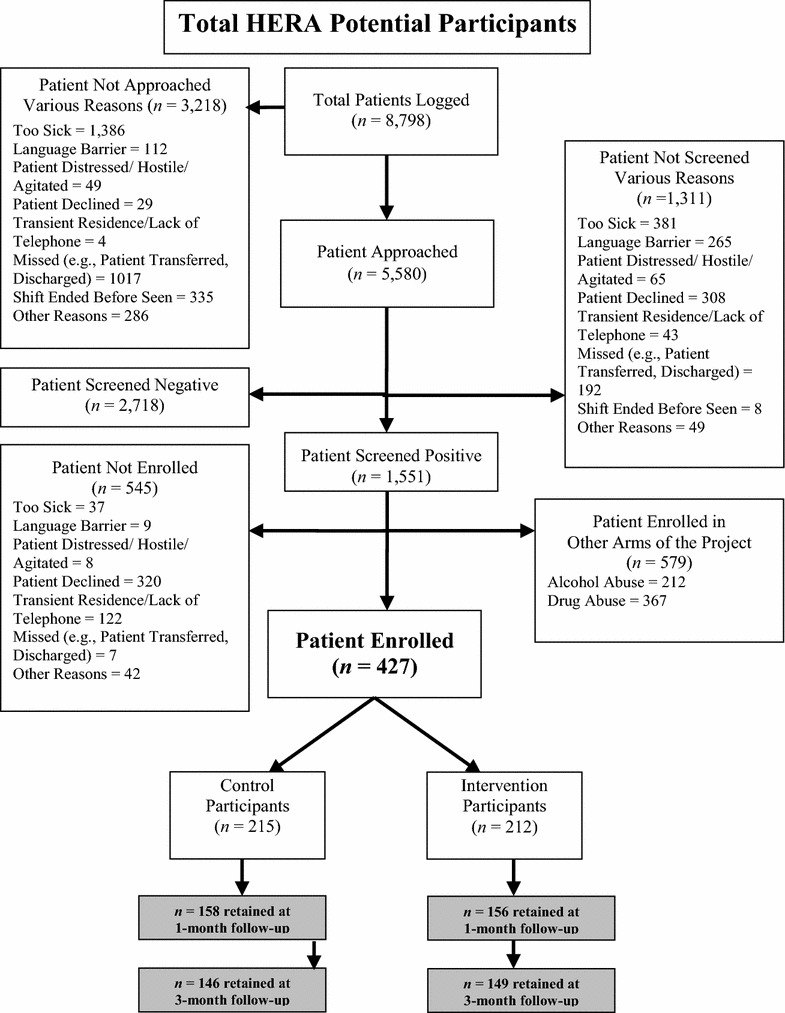
Enrollment flow for the HERA RCT—tobacco

Of 600 tobacco users who met eligibility criteria and who did not demonstrate risky alcohol and/or drug use, 427 individuals were enrolled (see Fig. 1). Those enrolled were not statistically different across demographics and enrollment site to those who were not enrolled, except enrolled individuals were slightly younger (*M* = 39.5 years; SD = 12.3) than non-enrolled individuals (*M* = 43.0 years; SD = 14.3), *t* (598) = 2.52, *p* = 0.01. Of the 427 participants enrolled, 215 were assigned to the control condition and 212 to the intervention. Six were eliminated due to either having been deceased before the follow-up assessment was complete or withdrawal from the study because of duplicate enrollment, resulting in an analyzed sample of 421 participants (211 Control, 210 HERA). At baseline, there were no differences between the two conditions on demographics, mental health diagnoses, HSI, pack years, or readiness to change (*p* values >0.10) (Table 2).

**Table 2:** Demographic characteristics of the analyzed sample

	Frequency	%	Mean	Standard deviation
Participant sex				
Male	150	35.6 %		
Female	271	64.4 %		
Participant age			39.0	12.2
Data collection site				
Cooper University Hospital (NJ)	233	55.3 %		
UMass University Hospital (MA)	178	42.3 %		
UMass Memorial Hospital (MA)	4	1.0 %		
Marlborough Hospital (MA)	6	1.4 %		
Race				
White	291	69.1 %		
Black	114	27.1 %		
Other/undocumented	16	3.8 %		
Ethnicity				
Non-hispanic/Latino	339	80.5 %		
Hispanic/Latino	67	15.9 %		
Not documented	15	3.6 %		
Insurance status^a^				
Private insurance	80	19.0 %		
Medicaid	127	30.2 %		
State financed insurance—non-medicaid	78	18.5 %		
Medicare	69	16.4 %		
Other kind of insurance not listed	27	6.4 %		
No insurance	73	17.3 %		
PHQ-2			1.7	1.7
Any mental health diagnosis	240	57.0 %		
Anxiety/panic attacks/PTSD	158	37.5 %		
Depression/bipolar disorder	150	35.6 %		
Schizophrenia/schizoaffective disorder	12	2.9 %		
Anorexia/bulimia	4	1.0 %		
ADD/ADHD	29	6.9 %		
Other mental health diagnosis	17	4.0 %		
Baseline HSI score			2.5	1.4
Pack years			18.2	14.9
Other tobacco used in past 30 days				
Cigars	14	3.3 %		
Smokeless tobacco (Dip, Snuff, Chew)	2	0.5 %		
Experimental condition				
Intervention	210	49.9 %		
Control	211	50.1 %		
Referral^b^				
Tailored, printed list of providers (only)	138	65.7 %		
Dynamic referral accepted	72	34.3 %		

Risky alcohol and illicit drug users were excluded from the tobacco sample; they were enrolled and randomized into the alcohol and drug use samples, to be reported separately

^a^Insurance status categories are not mutually exclusive (i.e. participants can have multiple types of insurance)

^b^Only participants in the intervention condition. All patients in the intervention group received the tailored, printed list of providers by default. Dynamic referral consists of a faxed referral with a brief tobacco use summary to a tobacco treatment provider matched to the individual based on location of residence and preference for telephone vs. in-person treatment. All patients in the intervention group received the tailored, printed list of providers by default

Of the 421 analyzed participants, 412 (98 %) completed the post-visit interview, 314 (75 %) completed the one-month follow-up, and 295 (70 %) completed the three-month follow-up (see Fig. 1). There were no differences between retained individuals and those lost to follow-up on intervention condition, most demographic characteristics, baseline HSI score, pack years, or readiness to change (*p* values >0.05); however, retained participants were slightly older (*M* = 39.8 years; SD = 12.3) than those lost to follow-up (*M* = 37.0 years; SD = 11.7), *t* (419) = 2.18, *p* = 0.03.

### Comparisons on outcomes of interest

*Specialized smoking cessation treatment* Initial contact between participants and a smoking cessation treatment provider was significantly more common in the intervention condition than the control condition (odds ratio = 2.69; see Table 3), with significant differences observed at each follow-up [1 month χ^2^ (1) = 8.16, *p* = 0.004; 3 months χ^2^ (1) = 11.65, *p* = 0.001]. Although treatment initiation, treatment engagement, and treatment completion rates were higher in the intervention condition compared to the control, these differences did not reach statistical significance (*p* values >0.05). Among the participants in the HERA, 72 (34 %) accepted a dynamic referral.

**Table 3:** Comparisons between intervention and control conditions

	Intervention (*n* = 210)	Control (*n* = 211)
ED clinician (MD/RN) counseling^a^		
MD/RN asked about tobacco use	172 (81.9 %)	165 (78.2 %)
MD/RN counseled participant to quit	73 (34.8 %)	68 (32.2 %)
Received educational materials	8 (3.8 %)	14 (6.6 %)
Received a smoking cessation referral	5 (2.4 %)	11 (5.2 %)
Outpatient smoking cessation treatment		
Contact with tobacco treatment provider GEE odds ratio = 2.69 (95 % CI 1.65–4.39), *p* < 0.001		
Contact at 1 month	35 (16.7 %)	16 (7.6 %)
Contact at 3 months	48 (22.9 %)	27 (12.8 %)
Initiated treatment (evaluated by tobacco treatment provider) GEE odds ratio = 1.69 (95 % CI 0.86–3.31), *p* = 0.13		
Treatment initiation at 1 month	10 (4.8 %)	8 (3.8 %)
Treatment initiation at 3 months	21 (10.0 %)	14 (6.6 %)
Treatment engagement at either time	8 (3.8 %)	7 (3.3 %)
Treatment completion	7 (3.3 %)	6 (2.8 %)
Smoking behavior		
Used tobacco (since ED visit) GEE odds ratio = 0.98 (95 % CI 0.62–1.55), *p* = 0.93		
Abstinent for first month (since visit)	13 (6.2 %)	19 (9.0 %)
Abstinent for 3 months (since visit)	8 (3.8 %)	9 (4.3 %)
At least one quit attempt at 1 month	68 (32.4 %)	72 (34.1 %)
At least one quit attempt at 3 months	105 (50.0 %)	118 (55.9 %)
Attempted to reduce use at 1 month	125 (59.5 %)	122 (57.8 %)
Attempted to reduce use at 3 months	150 (71.4 %)	154 (73.0 %)

All percentages and analyses use the ITT principal of worst outcome for missing values

^a^ED clinician behavior assessment included behaviors over and above the materials provided as part of the research study. All patients in both groups had tobacco assessed as part of the study and received a referral list. The control group received a pre-printed list, while the intervention group received a personally tailored list, as well as a dynamic referral if desired

*Tobacco use* Quit attempts, efforts to reduce smoking, HSI, and sustained abstinence at both follow-up periods were not statistically different across intervention and control conditions (see Table 3).

*Physician behavior* Clinician counseling, provision of educational materials, and provision of referrals, beyond those provided as part of the study protocol, were not statistically different across intervention and control conditions (see Table 3).

*Exploring the effect of dynamic referrals* Supplemental GEE analyses demonstrated large differences across groups on treatment contact. Using dummy codes (control condition as the reference), results indicated that experimental participants who accepted a dynamic referral made contact with a provider a much greater rate than control individuals (odds ratio = 11.89, 95 % CI 6.70–21.09, *p* < 0.001; see Table 4). Effects on treatment initiation were of relatively similar magnitude, with much higher rates of initiation among experimental participants who accepted a dynamic referral and control participants (odds ratio = 4.41, 95 % CI 2.11–9.21, *p* < 0.001). There were no differences in treatment initiation between experimental individuals who did not accept a dynamic referral and control individuals (odds ratio = 0.52, 95 % CI 0.18–1.49, *p* = 0.22). The differences in treatment initiation between experimental participants who accepted a dynamic referral and control participants remained significant (*p* values <0.05) when accounting for baseline readiness to change and baseline HSI in post hoc GEE models (see Table 5).

**Table 4:** Comparisons across intervention, tailored list only; intervention, dynamic referral; and control conditions

	Intervention-provider list (*n* = 138)	Intervention-dynamic referral (*n* = 72)	Control (*n* = 211)
Outpatient smoking cessation treatment			
Contact with tobacco treatment provider			
Contact at 1 month	3 (2.0 %)	32 (44.4 %)	16 (7.6 %)
Contact at 3 months	6 (4.3 %)	42 (58.3 %)	27 (12.8 %)
Initiated treatment (evaluated by tobacco treatment provider)			
Treatment initiation at 1 month	2 (1.4 %)	8 (11.1 %)	8 (3.8 %)
Treatment initiation at 3 months	3 (2.2 %)	18 (25.0 %)	14 (6.6 %)
Treatment engagement, either time	2 (1.4 %)	6 (8.3 %)	7 (3.3 %)
Treatment completion	2 (1.4 %)	5 (6.9 %)	6 (2.8 %)
Tobacco cessation			
Abstinent for first month (since visit)	10 (7.2 %)	3 (4.2 %)	19 (9.0 %)
Abstinent for 3 months (since visit)	6 (4.3 %)	2 (2.8 %)	9 (4.3 %)
At least one quit attempt at 1 month	47 (34.1 %)	21 (29.2 %)	72 (34.1 %)
At least one quit attempt at 3 months	72 (52.2 %)	33 (45.8 %)	118 (55.9 %)
Attempted to reduce use at 1 month	80 (58.0 %)	45 (62.5 %)	122 (57.8 %)
Attempted to reduce use at 3 months	98 (71.0 %)	52 (72.2 %)	154 (73.0 %)

**Table 5:** Follow-up GEE results with covariates

	Predictor	Odds ratio (95 % CI)	*p* value
Treatment contact	Heavy Smoking Index	1.13 (0.90–1.140)	0.29
	Readiness to quit	1.21 (0.84–1.75)	0.30
	Intervention (list) vs. control	0.61 (0.27–1.41)	0.25
	Intervention (DR) vs. control	10.96 (6.12–19.61)	<0.001
Treatment initiation	Heavy Smoking Index	1.16 (0.85–1.59)	0.34
	Readiness to quit	1.60 (0.87–2.94)	0.13
	Intervention (list) vs. control	0.61 (0.20–1.85)	0.38
	Intervention (DR) vs. control	3.74 (1.79–7.81)	<0.001
Abstinence	Heavy Smoking Index	0.90 (0.74–1.08)	0.26
	Readiness to quit	1.10 (0.83–1.45)	0.51
	Intervention (list) vs. control	1.06 (0.61–1.86)	0.82
	Intervention (DR) vs. control	0.82 (0.36–1.85)	0.62

*DR* dynamic referral

There was also a significant effect of group membership on engagement in cessation treatment, χ^2^ (1) = 6.60, *p* = 0.037 (see Table 4). While engagement was relatively infrequent across all groups, the rate of engagement for those accepting a dynamic referral was more than double the rate observed in the control condition (8.3 vs. 3.3 %; see Table 4). This effect, however, was no longer significant when baseline readiness to change tobacco use and HSI were included as covariates (odds ratio control vs. tailored list only = 0.78, 95 % CI 0.19–3.18, *p* = 0.73; odds ratio control vs. dynamic referral = 1.83, 95 % CI 0.58–5.76, *p* = 0.30). There were no effects of group membership on tobacco cessation (see Tables 4 and 5).

## Discussion

Providing tobacco interventions in the ED is important because many patients treated in EDs do not receive care anywhere else, which makes this setting important for comprehensive public health efforts to improve tobacco cessation [18]. However, there are unique barriers that make incorporating interventions into routine clinical care difficult, including competing priorities, time demands, acute care scope of practice, and a lack of clinician training related to smoking cessation interventions. As a result, technology facilitated interventions may be a particularly good fit. The results of this clinical trial studying a novel, single administration, stand-alone computerized intervention were mixed. Overall, the results suggested that the HERA may improve the critical step of initiating contact with an outpatient smoking cessation treatment provider after the ED visit, with the dynamic referral further promoting the next step of treatment initiation, but these effects do not translate into continued engagement in treatment or changes in smoking behavior over the 3 months following the ED visit. The low engagement rates of the HERA are consistent with rates from a similar study by Willet et al. to investigate the utility of faxed referrals to promote engagement with tobacco quitlines [19].

Factors that may have affected the HERA’s impact upon treatment and smoking cessation include lack of supportive efforts by ED staff, the low intensity of the HERA intervention, and barriers to patient follow through after contact with a smoking cessation treatment provider. It was deployed largely as a stand-alone intervention, because the practical realities of current ED practice suggested this would be the model most likely to be used in the ED environment. Although the clinicians who received the Healthcare Provider Report were educated on how to interpret the findings, and it could have prompted them to provide counseling to the patient, they were not specifically trained or mandated to provide counseling or any additional intervention materials. The analyses showed that, in fact, the intervention group did not receive any additional counseling or smoking cessation materials from their treating clinicians. A study of ED-based multicomponent intervention for tobacco use by Bernstein et al. [20] found that patients who received enhanced care in a variety of forms, including clinician counseling, nicotine patches, and follow-up phone call, were more likely to be abstinent at 3 months than those receiving standard referral. Greater clinician involvement may be needed to increase abstinence.

The HERA was a one-time interaction designed to be brief out of sensitivity to the time demands in the ED. This could have adversely affected its clinical impact. Future technology interventions may need to incorporate more powerful motivational interventions, such as interactive multi-media content, and longitudinal interaction after the ED visit. Longitudinal computerized interventions that provide personalized feedback reports over time have demonstrated evidence of effectiveness in several well-controlled trials, such as the Tobacco Expert System [21–23]. Blending the report generating and dynamic referral capabilities of the HERA with the longitudinal support and monitoring of systems like the Tobacco Expert System may represent the next evolution in technology facilitated tobacco interventions.

The HERA may not have promoted treatment initiation and engagement because other insurmountable barriers might have been present. For example, even for an individual who is highly motivated and successfully connects with a smoking cessation treatment provider, attending in-person counseling sessions can be impossible if he or she does not have transportation, has young children but no access to childcare, or works during hours when the services are available [7]. Moreover, some smoking cessation services charge a fee, which may have been prohibitive for some patients. Individuals were offered a toll-free telephone counseling option through the tobacco quitline 1-800-QUIT-NOW to help address cost and inconvenience barriers, but this may not have been sufficient to account for all barriers.

### Limitations

Several limitations affect interpretation of the findings. The study did not use biochemical validation of smoking abstinence. The impact on interpretation is likely to be minimal, because a treatment effect on smoking status was not observed. A minimal treatment control group was used, rather than true treatment as usual. The assessment and resource list may have acted as an intervention and artificially inflated treatment contact and behavior change compared to true treatment as usual. The study enrolled ED patients, which have been shown to change their smoking behavior after the ED visit naturalistically, especially when they perceived their visit to be smoking-related [7, 24–26]. It is particularly difficult to extend an intervention effect beyond this natural change motivator of the visit itself. Costs associated with the treatment services may have been a barrier to treatment initiation and engagement, but were partially mitigated by the inclusion of free treatment options in addition to the fee-for-service selections. Furthermore, some evidence suggests cost does influence follow-up with smoking cessation treatment referrals [27]. The study was also limited because participants were not questioned about why they failed to initiate or remain engaged with treatment after receiving the referral, and such barriers should be explored in future studies of this or similar systems. By focusing solely on tobacco users who do not abuse alcohol and/or drugs, the generalizability of our findings is somewhat limited. Future research should seek to examine the efficacy of automated referral generation systems for tobacco cessation among all tobacco users. Finally, the study follow-up period was limited to 3 months because of the restrictions associated with the NIH SBIR/STTR funding mechanism.

## Conclusion

The HERA helps to satisfy clinical practice mandates to Ask, Advise, and Refer tobacco users in the ED setting and was effective at promoting initial contact with a tobacco treatment provider. The dynamic referral option further promoted treatment initiation. However, the HERA, when deployed as a stand-alone intervention, did not lead to sustained post-visit tobacco treatment engagement or changes in smoking behavior within 3 months after the ED visit. The results raise the question of whether the Ask, Advise, and Refer method is adequate for all populations, particularly those discouraged by, or unable to pay, fees associated with treatment services or those with a lack of access to healthcare, which were represented by this study. The study highlights the importance of developing and studying interventions that work in conjunction with tobacco treatment linkage strategies.

## Abbreviations

SBIRT: screening, brief intervention, and referral to treatment
HERA: health evaluation and referral assistant
GEE: generalized estimating equation
ED: emergency department
RCT: randomized controlled trial
HSI: Heavy Smoking Index
RA: research assistant
